# Recognizing sinonasal cancer in primary care: a matched case–control study using electronic records

**DOI:** 10.1093/fampra/cmab153

**Published:** 2021-12-06

**Authors:** Tuba Khan, Yusera El-Sockary, William T Hamilton, Elizabeth A Shephard

**Affiliations:** 1 University of Exeter Medical School, St Luke’s Campus, Magdalen Road, Exeter, United Kingdom; 2 College of Medicine and Health, University of Exeter, College House, St Luke’s Campus, Exeter, United Kingdom

**Keywords:** diagnosis, early detection of cancer, general practice, nasopharynx, paranasal sinuses, primary health care

## Abstract

**Background:**

Cancers of the nasopharynx, nasal cavity, and accessory sinuses (“sinonasal”) are rare in England, with around 750 patients diagnosed annually. There are no specific National Institute for Health and Care Excellence (NICE) referral guidelines for these cancers and no primary care research published.

**Objective:**

To identify and quantify clinical features of sinonasal cancer in UK primary care patients.

**Methods:**

This matched case–control study used UK Clinical Practice Research Datalink (CPRD) data. Patients were aged ≥40 years with a diagnosis of sinonasal cancer between January 1, 2000 and December 31, 2009 and had consulted their GP in the year before diagnosis. Clinical features of sinonasal cancer were analysed using conditional logistic regression. Positive predictive values (PPVs) for single and combined features were calculated.

**Results:**

In total, 155 cases and 697 controls were studied. Nine symptoms and one abnormal investigation were significantly associated with the cancer: nasal mass; odds ratio, 95 (95% confidence interval 7.0, 1315, *P* = 0.001); head and neck lumps, 68 (12, 387, *P* < 0.001); epistaxis, 17 (3.9, 70, *P* < 0.001); rhinorrhoea, 14 (4.6, 44, *P* < 0.001); visual disturbance, 12 (2.2, 67, *P* = 0.004); sinusitis, 7.3 (2.2, 25, *P* = 0.001); sore throat, 6.0 (2.0, 18, *P* = 0.001); otalgia, 5.4 (1.6, 18, *P* = 0.007); headache, 3.6 (1.4, 9.5, *P* = 0.01); raised white cell count, 8.5 (2.8, 27, *P* < 0.001). Combined PPVs for epistaxis/rhinorrhoea, epistaxis/sinusitis, and rhinorrhoea/sinusitis were 0.62%.

**Conclusion:**

This is the first primary care study identifying epistaxis, sinusitis, and rhinorrhoea as part of the clinical prodrome of sinonasal cancer. Although no PPVs meet the 3% NICE referral threshold, these results may help clinicians identify who warrants safety-netting and possible specialist referral, potentially reducing the number of advanced-stage diagnoses of sinonasal cancer.

**Key messages**

- This is the first primary care study of the clinical prodrome of sinonasal cancer.
- Rhinorrhoea, headache, and epistaxis were most commonly reported.
- Nasal mass and head and neck lumps produced the highest positive predictive values (PPVs).
- All PPVs are below the NICE referral threshold.
- Patients who re-attend with these symptoms may warrant ENT referral.
- Earlier recognition of this rare cancer might improve survival outcomes.

## Background

Cancer of the nasopharynx, nasal cavity, and accessory sinuses are rare in the United Kingdom, accounting for <1% of all malignancies.^1,2^ In 2017, in England, 529 patients (60% male) were diagnosed with nasal cavity and accessory sinuses cancers: a further 218 patients (70% male) were diagnosed with nasopharyngeal cancer.^2^ The incidence of these cancers is less than 1 in 100,000^3,4^ and has remained static.^4^ The 5-year UK survival rate for both cancer sites is approximately 50%.^5^

Currently, there are no referral recommendations for these cancers within National Institute for Health and Care Excellence (NICE) guidelines.^6^ Referral guidelines for head and neck cancers only include specific recommendations for thyroid, larynx, and oral cancers. General practitioner (GP) referral is the commonest route to diagnosis (39%) for nasopharyngeal cancer, with those presenting as an emergency having the poorest survival outcomes.^7^ Previous secondary and tertiary care studies have reported multiple sinonasal disease symptoms including epistaxis,^8^ head and neck masses,^9^ nasal obstruction,^10^ rhinorrhoea,^11^ otalgia,^12^ headache,^13^ cranial nerve palsies, and visual disturbances.^14^ Unilateral sinonasal disease is more likely to be associated with malignancy than bilateral disease.^15^ Epistaxis and nasal obstruction are commonly reported^16^: in some cases, epistaxis predated diagnosis by 4 years.^17^ Symptoms of nasopharyngeal cancers include unilateral ear problems,^12^ cervical lymphadenopathy,^9^ and neck masses.^13^ This variation may be attributed to the disparate anatomical location of these cancers, with symptoms reflecting affected structures. These studies have combined head and neck cancer sub-sites differently. For sinonasal cancers, the mean duration of reported symptoms was approximately 6 months, with most presenting with advanced disease.^18^

The posterior nasal cavity, past the turbinates, is not easily visualized.^19^ This may contribute to diagnostic delay if the physician is unsure whether referral is warranted.^20^ Anterior rhinoscopy may be performed in primary care, but definitive endoscopic examination is reserved for specialist settings.^19^

The clinical prodrome of cancers of the nasopharynx, nasal cavity, and accessory sinuses (hereafter referred to as “sinonasal” cancer) has yet to be elucidated. The aim of this study was to identify the clinical features of sinonasal cancers in UK primary care patients, quantifying their individual and combined risk.

## Methods

This was a matched case–control study using primary care electronic patient records obtained from the UK’s Clinical Practice Research Datalink (CPRD). The CPRD collects anonymized patient records from 674 general practices.^21^ This database includes details of symptoms, investigations, diagnoses, and patient demographics.

### Cases and controls

A list of 20 nasopharyngeal and sinonasal cancer codes (ICD-10 codes C11, C30, C31) were collated from the CPRD master code library to identify relevant patients (specific READ codes available from authors upon request). “Read codes” are alphanumerical codes assigned to clinical terms. They are used in UK healthcare settings to identify symptoms, investigations, and treatments. It is similar to an electronic thesaurus for clinical terminology and allows you to search for the same term across different computer systems. Three codes were subsequently excluded as they involved the bony structure, cartilage, or unspecified area of the nose. Cases aged ≥40 years, diagnosed with sinonasal cancer between 2000 and 2009, were selected if they attended their GP in the year prior to diagnosis. Individuals aged 40+ were selected to reflect the age group where cancer incidence in the general population begins to rise. Each case was assigned up to five controls, matched via sex, age, and general practice. The date of diagnosis (“index date”) was determined by the first record of a sinonasal cancer code. Controls were matched to their respective case’s index date as per previous protocol.^22^ GP surgery was chosen as a proxy for socioeconomic status and to accommodate within-practice coding protocols. Exclusion criteria were: patients who had not consulted their GP in the year before the index date and cases without controls.

### Selection of putative clinical variables

Diagnostic features (clinical symptoms and investigations) were identified through literature searches using PubMed, Google Scholar, and ScienceDirect. Search terms included “nasopharyngeal/sinonasal cancer symptoms,” “early signs/indications/presentations of nasopharyngeal/sinonasal cancer,” and “primary care nasopharyngeal/sinonasal cancer.” Online forums were searched for any patient-reported symptoms of nasopharyngeal/sinonasal cancer prior to diagnosis. The CPRD master list contains over 100,000 medical codes mapped to READ codes. These correlate to individual clinical features and were assembled into feature-specific libraries, in line with our previous studies.^23,24^ Occurrence of these features in the year before the index date was recorded. Features present in ≥2% of cases were retained. Following our previous protocol, fractures were identified in both cases and controls to test for recording bias.^23,24^ Tests were considered abnormal if reported values fell outside the laboratory’s normal range. Patients within the expected ranges were grouped with those who had no test results.

### Composite variables

Thirteen composite symptom variables were generated: “hyposmia,” “catarrh,” “epiphora,” “facial pain,” “hearing loss,” “nasal mass,” “nasal septal ulcers,” “neck pain,” “ophthalmalgia,” “proptosis,” “rhinorrhoea,” “sinusitis,” and “tinnitus.” Hyposmia was comprised of agnosia, taste disorders, and complete anosmia. Catarrh included laryngitis, pharyngitis, tonsillitis, and infection. Nasal mass referred to both cysts and polyps. Nasal septal ulcers included perforation and necrosis. Rhinorrhoea was composed of rhinitis, nasal obstruction, and nasal discharge. The remaining variables contained codes with alternately worded derivatives and/or specified anatomical locations. Tests were combined to create the following groups: “raised inflammatory markers” (inclusive of plasma viscosity, erythrocyte sedimentation rate, and C-reactive protein) and “liver function tests” (measuring hepatic enzymes AST or ALT and bilirubin).

### Analysis and statistical methods

Conditional logistic regression was used for analysis. Individual features associated with sinonasal cancer with a *P*-value threshold of ≤0.1 in univariable analyses progressed to multivariable analysis. The final multivariable model used a *P*-value for retention of ≤0.01 and included clinically plausible interactions.

Positive predictive values (PPVs) for features of sinonasal cancer were generated using Bayes’ theorem (likelihood ratio multiplied by the prior odds of a feature equating to the posterior odds of having the cancer). The prior odds were determined by age-specific national incidence rates for sinonasal cancers in 2008. PPVs were only calculated for consulting patients aged 50+. PPVs were not calculated if <5 cases had the feature; when <10 cases or controls had the combined features, 95% CIs were omitted. PPVs for persistent symptoms were not calculated due to the limited number of cases. The posterior odds were subsequently divided by 0.9, based on 697 of a total 779 (89%) attending their GP in the previous year. The calculations in this study reflect the risk of an undiagnosed cancer based on symptom reporting by patients who physically present themselves to their GP. Thus 90% of the control population had the opportunity to report symptoms to their GP in person.

### Power calculation

The CPRD provided 177 cases and 884 controls. In accordance with our previous studies, power calculations were used in place of sample size calculations.^24,25^ The correlation between cases and controls was zero as both were assumed to have the same level of exposure. Using a case–control ratio of 1:5 with a 5% two-sided alpha produced >92% power to detect a change in common variables of 20% in cases and 10% in controls. All analyses used Stata/SE version 16.

## Results

**Fig. 1.**
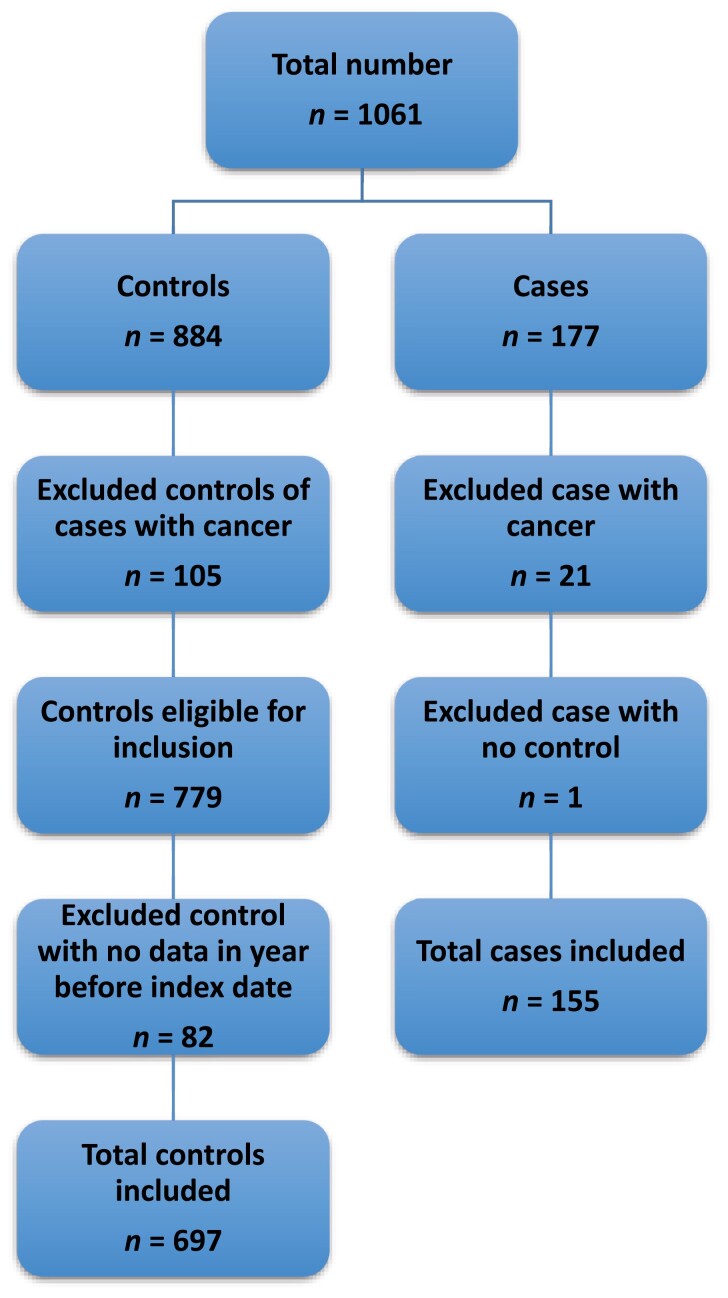
Exclusion criteria of 177 UK CPRD patients diagnosed with sinonasal cancer, 2000–2009 and 884 age-, sex-, and general practice-matched controls.

The CPRD supplied 1,061 patients (177 cases; 884 controls). Upon application of the exclusion criteria shown in Figure 1, a final number of 852 patients were included, (155 cases; 697 controls).

Patient demographic and consultation information is given in Table 1. Cases consulted significantly more frequently than controls in the year before diagnosis (*P* < 0.0001; rank-sum exact test).

**Table 1.** Characteristics and consultation rates of 155 UK patients, diagnosed with sinonasal cancer (2000–2009) and 697 matched controls.

	Cases			Controls		
	Male	Female	Total	Male	Female	Total
	(*n* = 102)	(*n* = 53)	(*n* = 155)	(*n* = 450)	(*n* = 247)	(*n* = 697)
Median (IQR) age in years at diagnosis	64	63	64	65	63	64
	(55–72)	(56–74)	(55–73)	(56–73)	(55–74)	(56–74)
Median (IQR) number of consultations in the 12-month preceding diagnosis	14	15	15^a^	7	8	7^a^
	(9–22)	(11–24)	(10–23)	(3–12)	(4–15)	(3–13)

Cases consulted significantly more frequently than controls in the year before diagnosis (*P* < 0.0001; rank-sum exact test).

### Clinical features

Thirty-two symptoms and fourteen investigations were considered initially; ten clinical features remained significant in the final model. The frequencies, univariable likelihood ratios, and multivariable odds ratios for these features are shown in Table 2. Sore throat and raised white cell counts were the only clinical features not previously reported in the secondary care literature but were present in our final model. From the 155 cases, 95 (61%) had at least one of the final model features recorded in the year preceding their diagnosis. The proportion of patients with a fracture did not differ between cases and controls (*P* < 0.580).

**Table 2.** Clinical features of sinonasal cancer in the UK patients aged ≥40 years: cases (*n* = 155) and controls (*n* = 697).

	Cases, *n* (%)	Controls, *n* (%)	Univariate likelihood ratio (95% CI)	Multivariate OR (95% CI)	*P*-value
Symptom					
Rhinorrhoea	28 (18)	11 (2)	12 (5.8–22)	14 (4.6–44)	<0.001
Headache	22 (14)	15 (2)	6.6 (3.5–12)	3.6 (1.4–9.5)	0.01
Epistaxis	19 (12)	6 (0.9)	14 (5.8–35)	17 (4.0–70)	<0.001
Sinusitis	18 (12)	9 (1)	9.0 (4.1–20)	7.3 (2.2–25)	0.001
Head and neck lumps	18 (12)	3 (0.4)	27 (8.1–90)	68 (12–388)	<0.001
Otalgia	13 (8)	8 (1)	7.3 (3.1–17)	5.3 (1.6–18)	0.007
Sore throat	10 (6)	12 (2)	3.8 (1.7–8.5)	6.0 (2.0–18)	0.001
Visual disturbances	9 (6)	2 (0.3)	20 (4.4–93)	12 (2.2–67)	0.004
Nasal mass	7 (5)	1 (0.1)	31 (3.9–254)	95 (7–1315)	0.001
Investigations					
Raised white cell count	14 (9)	14 (9)	4.5 (2.2–9.2)	8.5 (2.7–27)	<0.001

CI, confidence interval; OR, odds ratio.

### Positive predictive values

Single and combined PPVs were calculated for patients aged ≥50 years. The highest single PPV was 0.09% for head and neck lumps, well below the NICE referral threshold of 3%. The remaining PPVs were 0.06% for nasal polyps, 0.05% for visual disturbance, 0.04% for epistaxis, 0.03% for rhinorrhoea, 0.02% for otalgia, sinusitis, and headache, and 0.01% for sore throat and raised white cell count. The risk increased when patients reported more than one symptom; despite this, the highest risk was 0.62% for the following symptom combinations: epistaxis with rhinorrhoea, epistaxis with sinusitis, and rhinorrhoea with sinusitis. However, only 5 cases and no controls presented with these symptom combinations.

## Discussion

### Summary

This is the first study to identify and quantify the clinical features of sinonasal cancer in primary care. Features found to be associated with sinonasal cancer were similar to those reported in secondary care literature, with the exception of sore throat and a raised white cell count which are reported for the first time here. All single symptoms produced risk estimates of below 0.1%, reflecting the rarity of this cancer. The highest risk estimates were 0.62% for three pairs of symptoms: “epistaxis and rhinorrhoea,” “epistaxis and sinusitis,” and “rhinorrhoea and sinusitis.” Whilst these figures do not meet the 3% NICE threshold for urgent referral, they have now been identified as relevant primary care symptoms of sinonasal cancer and may warrant including as specific guidance in the next NICE cancer guideline update. With a lack of existing formal guidance, these results should assist GPs in their clinical decision making, particularly in patients with unresolved symptoms.

### Strengths and limitations

This is the first primary care study to investigate the clinical prodrome of sinonasal, accessory sinus, and nasopharyngeal cancers. Whilst these represent three distinct cancer sites, they have been grouped together as they follow a referral pathway to the same specialist. The results are therefore relevant for patient selection to secondary care. The study used the CPRD, which is representative of the UK population—ensuring generalisability of results. The quality and validity of primary care data collated by the CPRD have been well documented.^26^ This study was also adequately powered and the sample size was large, when compared to secondary care literature. The study period also involved direct transferral of laboratory results, reducing the likelihood of transcription errors. By reviewing current literature and patient forums, relevant features are unlikely to have been omitted. In calculating PPVs on the consulting population only, the results are directly applicable to clinical practice.

This study is reliant on coded symptom recording. Multiple codes may pertain to each symptom but a dominant generic code is usually selected. For two symptoms, nasal obstruction and epistaxis, we were not able to determine whether they were unilateral or bilateral symptoms. Although this study’s sample size was large compared to most secondary care studies, some symptoms combinations were not estimable due to low numbers. Similarly, caution should be advised in interpreting the results due to the wide confidence intervals.

Patients with sinonasal cancer consulted their GP more often than controls thereby having more chances to report symptoms. Relevant symptom information may be recorded in a “free-text” section which is inaccessible to researchers. This could result in missing data or an underestimation of symptom frequency, though this appears to be relatively minor.^27^ Bias from differential recording of symptoms appears to be relative to the under-recording of low-risk symptoms (like those seen in this study) in controls.^27^ If this bias has been present, our risk estimates, both odds ratios and PPVs may be slight under-estimates. There was no linkage to cancer registry data, therefore the stage at diagnosis for these patients is unknown.

### Comparison with existing literature

Sinonasal cancer patients consulted their GP on average twice as often as controls in the year preceding their diagnosis. This suggests that patients recognize a development of symptoms warranting medical appraisal—and provides the GP with opportunity to investigate. The features identified in this study mostly mirror those from secondary and tertiary care^1,8,10–14,17,18,28–30^; however, the frequency of symptom reporting varied. Epistaxis,^10,13,28^ headache,^13^ otalgia,^13^ and rhinorrhoea^10^ were reported both in secondary care and in this study. Secondary care reports from the United Kingdom identified palpable neck lumps as the most common symptom (55%), but most patients presented at advanced stages.^28^ Deafness/tinnitus was the second commonest feature,^28^ but this was not found in our primary care patient data. Similarly, cranial nerve palsies were only found in secondary care patients^9^; these differences may reflect the stage of cancer.^30^ Despite these findings, just under 40% of patients are not represented by these clinical features. It is likely that these patients reported features not common enough to be included, or those that failed to reach significance.

### Implications for practice

Current NICE guidelines for possible head and neck cancer only provide referral recommendations for larynx, thyroid, and oral cancers. There has been no primary care evidence base for sinonasal cancer until now. Although neither the single nor the combined PPVs of our final model features meet the 3% NICE urgent referral threshold, clinicians now know how this rare cancer may present in primary care. GPs may want to consider referral if a patient re-attends with persistent symptoms reported here, particularly multiple ones. The results of this study warrant including as a separate sub-site of head and neck cancers in future updates of NICE cancer guidelines.

## Conclusion

We have identified and quantified ten features associated with sinonasal cancer in primary care. Whilst the PPVs do not warrant immediate referral, patients with persistent features ought to be considered for further investigation. Clinical acumen and experience, which is difficult to quantify, may remain the most important factor in identifying patients with potential sinonasal cancer.^31^

## Supplementary Material

cmab153_suppl_Supplementary_Material
